# 3-Fluoro­salicylaldoxime at 6.5 GPa

**DOI:** 10.1107/S1600536809029043

**Published:** 2009-07-25

**Authors:** Peter A. Wood, Ross S. Forgan, Simon Parsons, Elna Pidcock, Peter A. Tasker

**Affiliations:** a Cambridge Crystallographic Data Centre, 12 Union Road, Cambridge CB2 1EZ, England; b School of Chemistry, The University of Edinburgh, King’s Buildings, West Mains Road, Edinburgh EH9 3JJ, Scotland

## Abstract

3-Fluoro­salicylaldoxime, C_7_H_6_FNO_2_, unlike many salicylaldoxime derivatives, forms a crystal structure containing hydrogen-bonded chains rather than centrosymmetric hydrogen-bonded ring motifs. Each chain inter­acts with two chains above and two chains below *via* π–π stacking contacts [shortest centroid–centroid distance = 3.295 (1) Å]. This structure at 6.5 GPa represents the final point in a single-crystal compression study.

## Related literature

For salicylaldoximes with bulky side groups which more commonly form hydrogen-bonded chains, see: Koziol & Kosturkiewicz (1984 ▶); Maurin (1994 ▶). For salicylaldoximes without bulky side groups that form chains, see: Wood *et al.* (2007*a* ▶,*b* ▶); Wood, Forgan, Parsons *et al.* (2006 ▶). For high pressure studies on salicyl­aldoximes, see: Wood *et al.* (2008 ▶, 2009 ▶); Wood, Forgan, Henderson *et al.* (2006 ▶). For specialized equipment used in the high pressure study, see: Merrill & Bassett (1974 ▶); Piermarini *et al.* (1975 ▶).
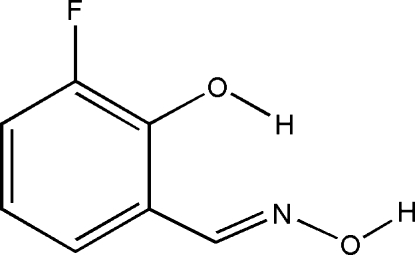


## Experimental

### 

#### Crystal data

C_7_H_6_FNO_2_
*M* *_r_* = 155.13
Orthorhombic, 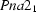

*a* = 6.156 (2) Å
*b* = 9.751 (3) Å
*c* = 8.6764 (18) Å
*V* = 520.8 (3) Å^3^
*Z* = 4
Mo *K*α radiation
μ = 0.17 mm^−1^
*T* = 298 K
0.15 × 0.12 × 0.10 mm

#### Data collection

Bruker APEXII diffractometer
Absorption correction: multi-scan (*SADABS*; Sheldrick, 2006 ▶) *T*_min_ = 0.39, *T*_max_ = 0.98
2715 measured reflections
333 independent reflections
233 reflections with *I* > 2σ(*I*)
*R*_int_ = 0.074

#### Refinement

*R*[*F* ^2^ > 2σ(*F* ^2^)] = 0.036
*wR*(*F* ^2^) = 0.049
*S* = 0.94
305 reflections
106 parameters
94 restraints
H atoms treated by a mixture of independent and constrained refinement
Δρ_max_ = 0.16 e Å^−3^
Δρ_min_ = −0.18 e Å^−3^

### 

Data collection: *APEX2* (Bruker, 2004 ▶); cell refinement: *SAINT* (Bruker, 2004 ▶); data reduction: *SAINT*; method used to solve structure: model taken from ambient pressure structure (Wood *et al.*, 2007*b* ▶); program(s) used to refine structure: *CRYSTALS* (Betteridge *et al.*, 2003 ▶); molecular graphics: *Mercury* (Macrae *et al.*, 2008 ▶); software used to prepare material for publication: *CRYSTALS*.

## Supplementary Material

Crystal structure: contains datablocks global, I. DOI: 10.1107/S1600536809029043/tk2511sup1.cif

Structure factors: contains datablocks I. DOI: 10.1107/S1600536809029043/tk2511Isup2.hkl

Additional supplementary materials:  crystallographic information; 3D view; checkCIF report

## Figures and Tables

**Table 1:** Hydrogen-bond geometry (Å, °)

*D*—H⋯*A*	*D*—H	H⋯*A*	*D*⋯*A*	*D*—H⋯*A*
O1—H1⋯O5^i^	0.89 (7)	1.81 (6)	2.684 (8)	165 (7)
O5—H5⋯N2	0.85 (4)	1.84 (6)	2.530 (7)	137 (6)

Symmetry code: (i) 
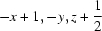
.

**Fig. 1.**
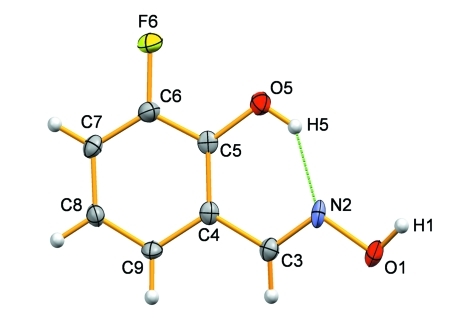
Molecular structure of (I) with probability ellipsoids drawn at the 50% level, showing atom labelling.

**Fig. 2.**
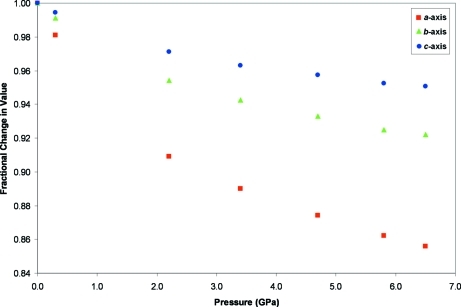
Graph of the fractional variation of the lattice parameters (a, b and c) of (I) as a function of pressure (GPa). The cell lengths are displayed as red squares, green triangles and blue circles for a, b and c, respectively.

**Fig. 3.**
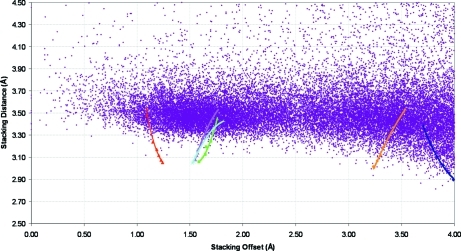
Graph of stacking distance (in Å) against stacking offset (in Å) for phenyl group stacking interactions in the CSD. The five previously published salicylaldoxime compression studies have been highlighted; salicylaldoxime (dark-blue), 3-chlorosalicylaldoxime (green), 3-methylsalicylaldoxime (pink), 3-methoxysalicylaldoxime (light-blue) and 3-tert-butylsalicylaldoxime (orange). Data for the compression study on (I) (red) has now also been added.

## supplementary crystallographic information

### Comment

Hydrogen-bonded chain formation is more common for salicylaldoximes with bulky side-groups, *e.g.* Koziol & Kosturkiewicz (1984) & Maurin (1994). The structure of the title compound, 3-fluorosalicylaldoxime, (I), which has been previously reported at ambient-pressure/150 K (Wood *et al.*, 2007*a*) is one of the exceptions to this trend along with salicylaldoxime-III (Wood, Forgan, Parsons *et al.*, 2006) and 3-hydroxysalicylaldoxime (Wood *et al.*, 2007*b*). Salicylaldoxime-I (Wood, Forgan, Henderson *et al.*, 2006) and four of its 3-substituted derivatives (Wood *et al.*, 2008), all of which form hydrogen-bonded dimers, have been studied under compression using a diamond-anvil high-pressure cell. This paper details the results of the continuation of the series to (I). The highest pressure structure is reported here but the ambient-pressure/ambient-temperature structure and further high pressure structures in between have been submitted to the CCDC as a private communication (Wood *et al.*, 2009).

Compound (I) crystallizes with one molecule in the asymmetric unit in the space group *Pna*2_1_ (Fig. 1). The molecule forms an intramolecular phenolic OH···N hydrogen bond [O5···N2 = 2.530 (7) Å] and an intermolecular oximic OH···O hydrogen bond [O1···O5 = 2.684 (8) Å] with a neighbouring molecule related by the 2_1_ screw axis. These two interactions taken together form a secondary level *C*(5) chain running parallel to the crystallographic *c* axis.

The compression of the 3-fluorosalicylaldoxime structure is anisotropic (Fig. 2). It can be seen that the *c*-axis is the least compressible (decreases by 4.9% up to 6.5 GPa) and this direction also corresponds to the direction of the hydrogen-bonded chains in the crystal structure. The next least compressible direction is that of the crystallographic *b* axis (decreases by 7.8%), where the main interactions are H···F and H···H contacts between the chains. Finally, the direction that compresses the most up to 6.5 GPa (14.4%) is along the *a* axis which corresponds to the π-π stacking direction; closest contact = Cg()···Cg()^i^ = 3.295 (1) Å for i: 0.5+x, 0.5-y, z.

Fig. 3 shows the compression of the π-π stacking contact geometry in comparison with equivalent interactions in other salicylaldoximes and with general stacking contacts found in the CSD. It can be seen that the compression follows the trend of the earlier oxime pressure studies (Wood *et al.*, 2008) in that the interaction compresses to the edge of what is seen at ambient conditions in the CSD. In this experiment the crystal disintegrated when the pressure was increased further and this may be indicative that a destructive phase transition occurred.

### Experimental

All solvents and reagents were used as received from Aldrich and Fisher. ^1^H and ^13^C NMR were obtained using a Bruker AC250 spectrometer at ambient temperature. Chemical shifts (δ) are reported in p.p.m. relative to internal standards. Fast atom bombardment mass spectrometry (FABMS) was carried out using a Kratos MS50TC spectrometer with a thioglycerol matrix. Analytical data was obtained from the University of St Andrews Microanalytical Service.

KOH (0.674 g, 10.20 mmol) and NH_2_OH.HCl (0.709 g, 10.20 mmol) were dissolved in EtOH, mixed thoroughly and a white KCl precipitate removed by filtration. 3-Fluorosalicylaldehyde (1.000 g, 7.14 mmol) was added to the filtrate, and the mixture refluxed for 3 h. The solvent was removed *in vacuo*, the residue redissolved in CHCl_3_, washed with water three times, and dried over MgSO_4_. The solvent was removed *in vacuo* to yield the crude product as a white powder (0.980 g, 88.5%). A pale-yellow block suitable for X-ray diffraction was grown by slow evaporation of a hexane/chloroform solution. (Anal. Calc. for C_7_H_6_FNO_2_: C, 54.2; H, 3.9; N, 9.0. Found: C, 54.3; H, 3.5; N, 9.2%); ^1^H NMR (250 MHz, CDCl_3_): δ(H) (p.p.m.) 6.78 (dt, 1H, ArH_b_), 6.90 (dd, 1H, ArH_a_), 7.05 (m, 1H, ArH_c_), 8.16 (s, 1H, CHN); ^13^C NMR (63 MHz, CDCl_3_) δ(C) (p.p.m.) 118.0 (aromatic CH), 118.7 (aromatic C-CHN), 119.8 (aromatic CH), 126.0 (aromatic CH), 145.9 (aromatic C—F), 152.8 (ArCHN), 153.6 (aromatic C—OH); FABMS *m*/*z* 156 (MH)+, 70%.

The high-pressure experiments were carried out using a Merrill-Bassett diamond anvil cell (half-opening angle 40°), equipped with brilliant-cut diamonds with 600 µm culets and a tungsten gasket (Merrill & Bassett, 1974). A 1:1 mixture of *n*-pentane and isopentane was used as a hydrostatic medium. Due to the high volatility of the *n*-pentane/isopentane solution, the cell was cooled in dry-ice prior to loading. A small ruby chip was also loaded into the cell as the pressure calibrant, with the ruby fluorescence method being used to measure the pressure (Piermarini *et al.*, 1975).

Following data collection, an absorption correction was applied using the program *SADABS* (Sheldrick, (2006). The T_max_/T_min_ ratio is larger than calculated on the basis of the crystal dimensions. However, multi-scan procedures (such as *SADABS* used in the present study) correct for all systematic errors that lead to disparities in the intensities of equivalent data. It is likely that the larger than expected range of transmission is accounted for by crystal decay or absorption by the high pressure cell.

### Refinement

The hydrogen atoms were located in a Fourier difference map. The positional and isotropic displacement parameters were then refined subject to restraints [C—H = 0.93 (2) Å, O—H = 0.82 (2) Å and *U*_iso_(H) = 1.5 *U*_eq_(C or O)]. In subsequent cycles of least-squares refinement all the *U*_iso_(H) values were fixed and the H-atoms attached to C were constrained to ride on their parent atoms. H1 and H5 were refined subject to distance restraints equal to 0.84 (5) Å.

In the absence of significant anomalous scattering effects, 316 Friedel pairs were averaged in the final refinement.

The crystal quality was beginning to deteriorate by this pressure and the number of reflections collected also dropped. In order to deal with this, global vibration and thermal similarity restraints were applied to the model.

### Crystal data

**Table:**

C_7_H_6_FNO_2_	*F*(000) = 320
*M**_r_* = 155.13	*D*_x_ = 1.978 Mg m^−^^3^
Orthorhombic, *P**n**a*2_1_	Mo *K*α radiation, λ = 0.71073 Å
Hall symbol: P 2c -2n	Cell parameters from 779 reflections
*a* = 6.156 (2) Å	θ = 3–24°
*b* = 9.751 (3) Å	µ = 0.17 mm^−^^1^
*c* = 8.6764 (18) Å	*T* = 298 K
*V* = 520.8 (3) Å^3^	Block, pale-yellow
*Z* = 4	0.15 × 0.12 × 0.10 mm

### Data collection

**Table:**

Bruker APEXII diffractometer	233 reflections with *I* > 2σ(*I*)
graphite	*R*_int_ = 0.074
ω scans	θ_max_ = 27.0°, θ_min_ = 3.1°
Absorption correction: multi-scan (*SADABS*; Sheldrick, 2006)	*h* = −6→6
*T*_min_ = 0.39, *T*_max_ = 0.98	*k* = −10→10
2715 measured reflections	*l* = −10→10
333 independent reflections	

### Refinement

**Table:**

Refinement on *F*^2^	Hydrogen site location: inferred from neighbouring sites
Least-squares matrix: full	H atoms treated by a mixture of independent and constrained refinement
*R*[*F*^2^ > 2σ(*F*^2^)] = 0.036	Method, part 1, Chebychev polynomial, (Watkin, 1994, *P*rince, 1982) [*w*eight] = 1.0/[A_0_*T_0_(x) + A_1_*T_1_(x) ··· + A_n-1_]*T_n-1_(x)] where A_i_ are the Chebychev coefficients listed belo*w* and x = *F* /*F*max Method = Robust Weighting (*P*rince, 1982) W = [*w*eight] * [1-(delta*F*/6*sigma*F*)^2^]^2^ A_i_ are 12.4 13.8 4.45 -0.254
*wR*(*F*^2^) = 0.049	(Δ/σ)_max_ < 0.001
*S* = 0.94	Δρ_max_ = 0.16 e Å^−^^3^
305 reflections	Δρ_min_ = −0.18 e Å^−^^3^
106 parameters	Extinction correction: Larson (1970), Equation 22
94 restraints	Extinction coefficient: 119.189
Primary atom site location: structure-invariant direct methods	

### Fractional atomic coordinates and isotropic or equivalent isotropic displacement parameters (Å^2^)

**Table:**

	*x*	*y*	*z*	*U*_iso_*/*U*_eq_	
O1	0.4779 (9)	0.1918 (6)	0.9058 (5)	0.0268	
N2	0.4588 (11)	0.1849 (7)	0.7440 (5)	0.0247	
C3	0.4049 (11)	0.3001 (7)	0.6852 (7)	0.0191	
C4	0.3841 (12)	0.3034 (7)	0.5185 (6)	0.0157	
C5	0.3721 (11)	0.1828 (9)	0.4328 (5)	0.0143	
O5	0.3858 (9)	0.0571 (5)	0.4956 (5)	0.0214	
C6	0.3455 (12)	0.1950 (9)	0.2755 (6)	0.0201	
F6	0.3356 (7)	0.0740 (5)	0.1972 (4)	0.0265	
C7	0.3246 (12)	0.3167 (8)	0.2001 (7)	0.0185	
C8	0.3431 (12)	0.4332 (8)	0.2861 (6)	0.0189	
C9	0.3738 (11)	0.4309 (8)	0.4436 (6)	0.0183	
H3	0.3781	0.3782	0.7440	0.0230*	
H7	0.3014	0.3204	0.0958	0.0219*	
H8	0.3343	0.5184	0.2363	0.0227*	
H9	0.3877	0.5129	0.4979	0.0218*	
H5	0.392 (14)	0.059 (7)	0.594 (4)	0.0321*	
H1	0.546 (15)	0.114 (6)	0.929 (7)	0.0400*	

### Atomic displacement parameters (Å^2^)

**Table:**

	*U*^11^	*U*^22^	*U*^33^	*U*^12^	*U*^13^	*U*^23^
O1	0.037 (5)	0.032 (5)	0.0119 (13)	0.002 (2)	0.0009 (19)	0.003 (2)
N2	0.035 (6)	0.028 (4)	0.0109 (15)	−0.001 (3)	0.001 (2)	0.008 (3)
C3	0.017 (5)	0.022 (4)	0.0176 (17)	0.000 (3)	0.001 (3)	0.001 (2)
C4	0.010 (4)	0.020 (2)	0.0176 (18)	0.001 (2)	−0.002 (3)	0.0018 (16)
C5	0.004 (4)	0.020 (2)	0.0189 (19)	0.000 (2)	0.000 (3)	0.0014 (16)
O5	0.023 (4)	0.022 (2)	0.019 (2)	0.0014 (19)	0.003 (2)	0.0014 (18)
C6	0.022 (6)	0.020 (2)	0.019 (2)	0.000 (3)	−0.001 (3)	−0.0035 (16)
F6	0.035 (3)	0.023 (2)	0.0215 (17)	0.0051 (16)	0.001 (2)	−0.0071 (18)
C7	0.017 (5)	0.024 (3)	0.015 (2)	0.002 (2)	0.002 (3)	−0.0008 (18)
C8	0.022 (5)	0.017 (3)	0.018 (2)	0.000 (2)	0.001 (3)	0.004 (2)
C9	0.022 (5)	0.016 (3)	0.017 (2)	0.005 (2)	0.002 (3)	−0.003 (2)

### Geometric parameters (Å, °)

**Table:**

O1—N2	1.410 (6)	O5—H5	0.86 (4)
O1—H1	0.89 (4)	C6—F6	1.363 (9)
N2—C3	1.278 (9)	C6—C7	1.361 (9)
C3—C4	1.452 (8)	C7—C8	1.365 (10)
C3—H3	0.932	C7—H7	0.917
C4—C5	1.394 (9)	C8—C9	1.380 (8)
C4—C9	1.404 (10)	C8—H8	0.938
C5—O5	1.345 (9)	C9—H9	0.932
C5—C6	1.380 (7)		
			
N2—O1—H1	103 (4)	C5—C6—F6	115.1 (7)
O1—N2—C3	112.2 (7)	C5—C6—C7	124.2 (7)
N2—C3—C4	116.2 (7)	F6—C6—C7	120.8 (5)
N2—C3—H3	123.1	C6—C7—C8	117.1 (6)
C4—C3—H3	120.8	C6—C7—H7	121.6
C3—C4—C5	121.2 (6)	C8—C7—H7	121.3
C3—C4—C9	119.0 (6)	C7—C8—C9	122.6 (8)
C5—C4—C9	119.8 (5)	C7—C8—H8	118.8
C4—C5—O5	123.3 (4)	C9—C8—H8	118.6
C4—C5—C6	117.5 (7)	C4—C9—C8	118.6 (7)
O5—C5—C6	119.1 (7)	C4—C9—H9	121.4
C5—O5—H5	113 (5)	C8—C9—H9	119.9

### Hydrogen-bond geometry (Å, °)

**Table:**

*D*—H···*A*	*D*—H	H···*A*	*D*···*A*	*D*—H···*A*
O1—H1···O5^i^	0.89 (7)	1.81 (6)	2.684 (8)	165 (7)
O5—H5···N2	0.85 (4)	1.84 (6)	2.530 (7)	137 (6)

Symmetry codes: (i) −*x*+1, −*y*, *z*+1/2.

## References

[bb1] Betteridge, P. W., Carruthers, J. R., Cooper, R. I., Prout, K. & Watkin, D. J. (2003). *J. Appl. Cryst.* **36**, 1487.

[bb8] Bruker (2004). *APEX2* and *SAINT* Bruker AXS Inc., Madison, Wisconsin, USA.

[bb2] Koziol, A. E. & Kosturkiewicz, Z. (1984). *Pol. J. Chem.* **58**, 569–575.

[bb3] Macrae, C. F., Bruno, I. J., Chisholm, J. A., Edgington, P. R., McCabe, P., Pidcock, E., Rodriguez-Monge, L., Taylor, R., van de Streek, J. & Wood, P. A. (2008). *J. Appl. Cryst.* **41**, 466–470.

[bb4] Maurin, J. K. (1994). *Acta Cryst.* C**50**, 1357–1359.

[bb5] Merrill, L. & Bassett, W. A. (1974). *Rev. Sci. Instrum.* **45**, 290–294.

[bb6] Piermarini, G. J., Block, S., Barnett, J. D. & Forman, R. A. (1975). *J. Appl. Phys.* **46**, 2774–2780.

[bb7] Sheldrick, G. M. (2006). *SADABS* University of Göttingen, Germany.

[bb10] Wood, P. A., Forgan, R. S., Henderson, D., Parsons, S., Pidcock, E., Tasker, P. A. & Warren, J. E. (2006). *Acta Cryst.* B**62**, 1099–1111. 10.1107/S0108768106031752 17108665

[bb11] Wood, P. A., Forgan, R. S., Lennie, A. R., Parsons, S., Pidcock, E., Tasker, P. A. & Warren, J. E. (2008). *CrystEngComm*, **10**, 239–251.

[bb12] Wood, P. A., Forgan, R. S., Parsons, S., Pidcock, E. & Tasker, P. A. (2006). *Acta Cryst.* E**62**, o3944–o3946. 10.1107/S0108768106031752 17108665

[bb13] Wood, P. A., Forgan, R. S., Parsons, S., Pidcock, E. & Tasker, P. A. (2007*a*). *Acta Cryst.* E**63**, o3132.

[bb14] Wood, P. A., Forgan, R. S., Parsons, S., Pidcock, E. & Tasker, P. A. (2007*b*). *Acta Cryst.* E**63**, o3131.

[bb15] Wood, P. A., Forgan, R. S., Parsons, S., Pidcock, E. & Tasker, P. A. (2009). Private communication to the Cambridge Structural Database (deposition numbers CCDC 737481–737486). CCDC, Union Road, Cambridge, England.

